# Metal levels in bat guano from Brazilian caves: insights into environmental contamination

**DOI:** 10.1007/s10661-026-15456-1

**Published:** 2026-05-23

**Authors:** Karla Beatriz Oliveira da Silva, Narjara Tércia Pimentel, Eder Barbier, Enrico Bernard, Ana Paula Silveira Paim

**Affiliations:** 1 Departamento de Química Fundamental, Universidade Federal de Pernambuco, Recife, Pernambuco, Brazil; 2 Departamento de Zoologia, Universidade Federal de Pernambuco, Recife, Pernambuco Brazil; 3 Centro de Ciências Biológicas, Universidade Estadual do Norte do Paraná, Bandeirantes, Paraná Brazil; 4 Departamento de Ecologia e Conservação, Instituto de Ciências Naturais, Universidade Federal de Lavras, Lavras, Minas Gerais Brazil

**Keywords:** Chiroptera, Bioindicators, ICP OES, Mammals, Bioaccumulation

## Abstract

Bats, with more than 1450 species worldwide, roost in caves, and colonies can produce guano deposits. Guano analysis reveals bat diets and indicates environmental contamination, since bats interact and accumulate metals through food, water, and airborne particulate matter. This study examined guano from caves in Rio Grande do Norte (RN-27 samples) and Pará (PA-24 samples), Brazil, to assess metal levels. Samples were taken from different depths, locations, and species, including the omnivorous Phyllostomus discolor, insectivorous Pteronotus gymnonotus, and nectarivorous Glossophaga soricina. Microwave-assisted acid digestion was used, and a 2^3^ factorial design optimized acid digestion conditions based on residual acidity (RA) and dissolved organic carbon (DOC). Optimal parameters were 10.0 mL of 1.5 mol L⁻^1^ HNO₃, 3.0 mL H₂O₂, and 35 min digestion. Cd, Co, Cr, and Pb were detected in samples from RN, with concentrations reaching 2.3, 5.2, 18.6, and 30.7 mg kg⁻^1^. In PA, Pb concentrations of 20.0 and 19.4 mg kg⁻^1^ were observed in two samples, and Cd concentrations ranged from 0.2 to 2.3 mg kg⁻^1^. By contrast, Cu was detected at concentrations ranging from 64.9 to 283.0 mg kg⁻^1^ in RN and 10.4 to 645.6 mg kg⁻^1^ in PA. Such levels likely reflect bioaccumulation, as Cu is an essential nutrient, but are not explained solely by physiological regulation and may also reflect environmental conditions at the sampling sites, while Pb indicates environmental exposure. These findings highlight the need to monitor metal contamination, with bat guano proving a useful indicator.

**Graphical abstract:**

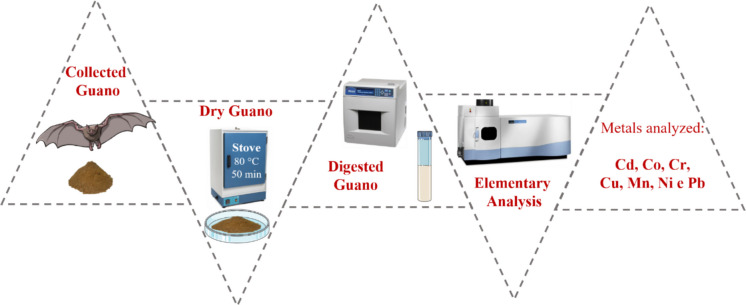

**Supplementary Information:**

The online version contains supplementary material available at 10.1007/s10661-026-15456-1.

## Introduction

With around 1500 species distributed worldwide, bats (Chiroptera) are animals with ecological interactions at various trophic levels (Sakoui et al., 2020). Insectivorous bats, for example, are predators of thousands of species, including some pathogenic insects that threaten agricultural production and forest economies (Tuneu-corral et al., 2023). Such service as insect pest suppressors can save billions of dollars annually for farmers (Boyles et al., 2011). In Brazil, for example, the reduction in pesticide application costs in maize plantations, resulting from insect predation by bats, has been estimated to save approximately USD 390.6 million per year (Aguiar et al., 2021). Frugivorous and nectarivorous bats are also essential for forest biodiversity conservation, as they contribute to seed dispersal across various ecosystems (Cherobim et al., 2021; Ramíres-Fráncel et al., 2022). This process not only promotes the regeneration of native plant species but also facilitates the introduction of new species, therefore enhancing the dynamics and resilience of forest habitats (Sakoui et al., 2020).

However, despite their significant environmental benefits, bats face numerous threats, most of which are associated with human population growth (Kerth & Wolf, 2025; Mickleburgh et al., 2002; Souza et al., 2020). These mammals are exposed to a variety of pollutants, including toxic substances such as toxic metals, which pose substantial risks to their health and survival (Russo & Ancillotto, 2015). Such toxic metal emissions reach the environment through multiple pathways, including air pollution via combustion processes and the contamination of water and soil, adversely affecting vegetation, insects, and crops in the impacted regions (Zukal et al., 2015). Consequently, due to their varied diet, which includes insects, fruits, and nectar, bats are susceptible to the bioaccumulation of toxic metals in different organs, such as the liver and kidneys (Timofieieva et al., 2024; Vidal et al., 2024; Walker et al., 2007). Bat feces, known as guano, are often used in studies of regional contamination, making it possible to monitor environmental pollutants without sacrificing animals (Gonkowski et al., 2024; Madhappan et al., 2021; Martín et al., 2023). Since the dietary diversity of bats influences the composition of guano, it can also provide valuable insights into their foraging and roosting areas (Soliman et al., 2019; Zukal et al., 2015). Therefore, guano serves as an important bioindicator of environmental contamination.

The guano from insectivorous bats frequently contains potassium, ammonium, sodium, magnesium, and calcium phosphates and sulfates, but also phosphorus, aluminum, and iron in its surface layers (Fernández-Ibáñez et al., 2003; Lundberg & Mcfarlane, 2021; Piló et al., 2023; Sakthi & Jeyapraba, 2023). The presence of essential trace elements (e.g., Cu, Mn, Ni), which may become toxic at elevated concentrations, as well as non-essential toxic metals (e.g., Pb, Cd) has been identified in various regions around the world (Gallant et al., 2020; Wurster et al., 2015). The bioaccumulation of these trace elements can lead to adverse health impacts on bats, causing physiological and behavioral changes, and studies have shown that exposure to high levels of toxic metals can induce oxidative stress, cause liver and kidney damage, and lead to genotoxic effects (Bayat et al., 2014; Mickleburgh et al., 2002).

Brazil is a country with a rich bat fauna (186 species; Garbino et al., (2024)), but it also experiences high levels of habitat loss and degradation, particularly in the North and Northeast regions, where agriculture and mining are major drivers of environmental change (ICMBio 2016; MapBiomas, 2025). The selection of caves in these regions is justified by the presence of anthropogenic activities, such as intensive agriculture and mineral extraction, which may act as potential sources of metals. In addition, beyond anthropogenic inputs, natural geochemical variations related to the local lithology of cave systems may influence baseline trace element levels in guano. In Brazil, only a limited number of bat species have been investigated for toxic metal accumulation (Souza et al., 2020), highlighting the need for further studies encompassing additional species, regions, and sample types, as well as for analytical approaches capable of reliably characterizing metal concentrations in complex environmental matrices such as guano.

In this study, we aimed to evaluate the potential of bats as bioindicators by investigating the concentrations of key metals present in their guano sampled in caves in northern and northeastern Brazil. To achieve this, optimized conditions for microwave-assisted acid digestion were established, and the elemental composition was determined using inductively coupled plasma optical emission spectrometry (ICP OES).

## Materials and methods

### Study area

**Fig. 1:**
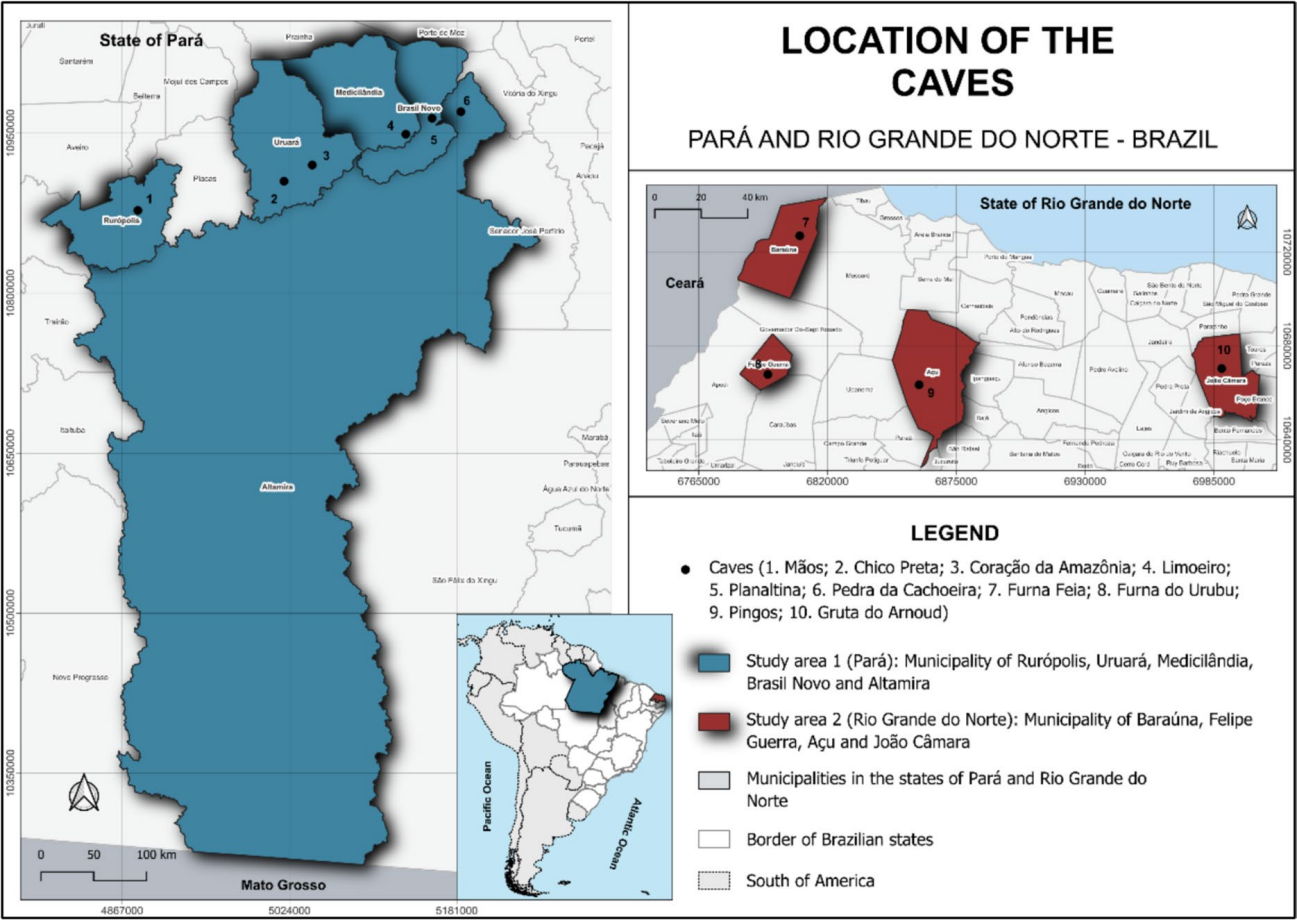
Study areas and location of the ten caves in the North (Pará) and Northeast (Rio Grande do Norte) regions of Brazil

Guano samples in this study were collected from ten caves located in two Brazilian states, Pará (PA) and Rio Grande do Norte (RN), situated in the North and Northeast regions of Brazil, respectively (Fig. 1). In Pará, six caves are distributed across the municipalities of Rurópolis, Uruará, Medicilândia, Brasil Novo, and Altamira, whereas in Rio Grande do Norte, the four sampled caves are located in the municipalities of Baraúna, Felipe Guerra, Açu, and João Câmara (Table 1).

**Table 1:** Location, physical characteristics (rock types and number of entrances) and the total number of bat species present in the 10 caves in Northern and Northeastern Brazil. PA — Pará, RN — Rio Grande do Norte

Cave	Municipality, State	Coordenates		Lithology	Entrances	Number of bats
		Latitude	Longitude			
Mãos	Rurópolis, PA	04º09`24.0″S	55º04`19.4″W	Sandstone	4	7
Chico Preta	Uruará, PA	03º54`46.1″S	53º49`49.3″W	Sandstone	2	8
Coração da Amazônia	Uruará, PA	03º46`31.0″S	53º35`24.7″W	Sandstone	1	8
Limoeiro	Medicilândia, PA	03º30`43.4″S	52º47`50.7″W	Sandstone	3	8
Planaltina	Brasil Novo, PA	03º22`39.8″S	52º34`29.7″W	Sandstone	1	8
Pedra da Cachoeira	Altamira, PA	03º19`14.0″S	52º19`54.9″W	Sandstone	4	12
Furna Feia	Baraúna, RN	05°02′11.1″S	37°33′36.8″W	Limestone	4	13
Furna do Urubu	Felipe Guerra, RN	05°34′22.8″S	37°39′08.8″W	Limestone	1	8
Pingos	Açu, RN	05°34′41.6″S	37°03′47.5″W	Limestone	2	1
Gruta do Arnoud	João Câmara, RN	05°26′36.2″S	35°53′37.1″W	Limestone	1	3

### Bat guano samples

A total of 27 guano samples were collected from Rio Grande do Norte, specifically from the caves Furna Feia (9 samples), Furna do Urubu (13 samples), Gruta do Arnoud (2 samples), and Gruta dos Pingos (3 samples). Twenty-four samples were collected in Pará, from Caverna das Mãos (6 samples), Chico Preta (3 samples), Coração da Amazônia (5 samples), Limoeiro (5 samples), Planaltina (4 samples) and Pedra da Cachoeira (1 sample) caves.

Guano samples were collected from different bat species and stratifications within the caves (Table 2). A total of 51 samples were obtained, of which 43 (84.3%) corresponded to surface deposits and 8 (15.7%) to stratified deposits from Furna do Urubu Cave (RN), the only site with sufficiently deep guano deposits (up to 150 cm). Surface sampling prioritized areas with recent deposition, identified during daytime surveys by the presence of active bat roosts, with guano collected directly beneath these colonies. In cases where direct observation of active roosts was not possible, recent deposits were selected based on physical characteristics, including darker coloration, higher moisture content, lack of desiccation, and the absence of visible fungal growth. From these areas, ~100 g of guano was collected after removing the exposed layer. In Furna do Urubu, additional samples were obtained from a stratigraphic profile previously excavated for paleoclimate studies (Utida et al., 2020), where guano was collected in successive layers, in smaller amounts, to avoid mixing between strata. All samples were collected with disposable plastic spoons, stored in hermetically sealed plastic bags, and labeled according to bat species, feeding guild, and collection depth (surface or stratigraphic). To confirm bat species identity, individuals roosting above sampling sites were captured with hand nets and identified following (López-Baucells et al., 2016) and (Diaz et al., 2021). After collection, samples were refrigerated (5 ± 1 °C) until analysis, with storage times ranging from 1 to 4 months.

**Table 2:** Location, bat species and trophic guild, number of samples, and depth of collection from caves in the states of Rio Grande do Norte (RN) and Pará (PA), Brazil

Cave (State)	Bat species	Samples and trophic guild	ID	Guano depth (in cm)
Furna Feia (RN)	*Phyllostomus discolor*	2 Omnivorous	RN-01 and RN-22	Superficial layer
	*Phyllostomus discolor*	1 Insectivorous	RN-18	
	*Pteronotus gymnonotus*	6 Insectivorous	RN-08, RN-11, RN-13, RN-16, RN-19 and RN-21	
Furna do Urubu (RN)	*Pteronotus gymnonotus*	8 Insectivorous	RN-03, RN-05, RN-06, RN-09, RN-17, RN-25, RN-26 and RN-27	12-14; 28-30; 44-46; 58-60; 76-78; 96-98; 120-122; and 148-150
		5 Insectivorous	RN-10, RN-14, RN-15, RN-23 and RN-24	Superficial layer
Gruta de Arnoud (RN)	*Pteronotus gymnonotus*	1 Nectarivorous	RN-12	Superficial layer
		1 Insectivorous	RN-20	
Gruta dos Pingos (RN)	*Pteronotus gymnonotus*	3 Insectivorous	RN-02, RN-04 and RN-07	Superficial layer
Caverna das Mãos (PA)	*Carollia perspicillata*	1 Frugivorous	PA-01	Superficial layer
	*Carollia perspicillata, Lonchophyllinae*	1 Frugivorous + Nectarivorous	PA-02	Superficial layer
	*Natalus macrourus, Pteronotus rubiginosus/P. alitonus*	4 Insectivorous	PA-03, PA-04, PA-05 and PA-06	Superficial layer
Chico Preta (PA)	*Natalus macrourus, Pteronotus rubiginosus/P. alitonus*	2 Insectivorous	PA-07 and PA-08	Superficial layer
	*Carollia perspicillata*	1 Frugivorous	PA-09	
Coração da Amazônia (PA)	*Natalus macrourus, Pteronotus rubiginosus/P. alitonus*	2 Insetívorous	PA-10 and PA-11	Superficial layer
	*Phyllostomus hastatus*	2 Omnivorous	PA-12 and PA-13	
	*Carollia perspicillata*	1 Frugivorous	PA-14	
Limoeiro (PA)	*Natalus macrourus, Pteronotus rubiginosus/P. alitonus*	3 Insectivorous	PA-15, PA-16 and PA-17	Superficial layer
	*Phyllostomus hastatus*	2 Omnivorous	PA-18 and PA-19	
Planaltina (PA)	*Pteronotus* sp.	1 Omnivorous	PA-20	Superficial layer
	*Phyllostomus hastatus* *Natalus macrourus*	2 Omnivorous	PA-21 and PA-22	
		1 Insectivorous	PA-23	
Pedra da Cachoeira (PA)	*Pteronotus* sp.	1 Insectivorous	PA-24	Superficial layer

### Analytical procedures

#### Instruments

The inductively coupled plasma optical emission spectrometer (ICP OES) (Optima 7000 DV model, PerkinElmer, Waltham-Massachusetts, USA) was used in axial configuration, with a solid-state CCD detector system, an argon-purged optical system, and Echelle optics. The wavelength range was from 160 to 900 nm, and the sample introduction system utilizes a Scott chamber and a cross-flow nebulizer. The wavelengths of the analyzed metals are listed in Table S1 (Supplementary Material).

Microwave-assisted acid digestions were carried out in a microwave radiation oven (model MARS Xpress, CEM Corporation, North Carolina, USA), with a temperature of 150 °C, a maximum power of 1600 W, a heating ramp and plateau of 35 min, and a cooling time of 20 min.

#### Microwave-assisted acid digestion

Preliminary tests to establish the digestion conditions were performed using a guano sample collected from Gruta do Arnoud, located in RN. The tests were realized to minimize the concentration of nitric acid (HNO_3_) required for the effective decomposition of guano. To achieve this, HNO_3_ concentrations of 7.0, 3.5, 2.0, and 1.0 mol L⁻^1^ were tested, while maintaining a constant volume of H_2_O_2_ (35%, v/v) at 2 mL. The efficiency of the digestion was initially assessed through visual inspection, with the successful decomposition indicated by the formation of a clear residue-free solution.

For each test, approximately 1.0 g of guano was first heated at 80 °C for 40 min and subsequently macerated. After drying, a 0.25 g portion of the resulting material (dry mass) was weighed and transferred into a digestion tube made of PTFE (Teflon®), with screw-cap closure and capable of withstanding high temperatures and internal pressures, followed by the addition of 10 mL of HNO_3_ (at the specified concentration) and 2 mL of H_2_O_2_. The conditions for each decomposition experiment are listed in Table S2 (Supplementary Material).

#### Validation method

The method was validated by evaluating the following performance parameters: limits of detection (LOD) and quantification (LOQ), as well as accuracy by analysis of a certified reference material (CRM, Solo RM-Agro E2002a, CENA). The LOD and LOQ were determined according to (INMETRO, 2020) guidelines, based on successive blank measurements and the standard deviation (s), using the expressions LOD = 3.3 s/b and LOQ = 10 s/b, where *b* is the slope of the calibration curve.

Co, Ni, and Zn were not reported in the CRM certificate; however, the elements were validated for accuracy by recovery tests. These experiments were carried out at three concentration levels (2.5, 5.0, and 7.5 mg L⁻^1^) using a guano sample.

#### Elemental analysis of digested guano

Metal concentrations were determined using ICP OES, using a calibration curve ranging from 0.1 to 4 mg L⁻^1^ Cadmium (Cd), Cobalt (Co), Chromium (Cr), Copper (Cu), Nickel (Ni) and Lead (Pb). The metal standard solutions were prepared by appropriate dilution of a 10 mg L^−1^ intermediate multi-element solution, which was obtained from a 1000 mg L^−1^ multi-element standard solution (Inorganic Ventures, Christiansburg-VA, USA). Analytical blanks were prepared using the same digestion matrix as the samples, consisting of 10 mL of HNO₃ (at the specified concentration) and 2 mL of H₂O₂, but without the addition of guano, in order to evaluate potential contamination from reagents and analytical procedures.

### Statistical analyses

#### Factorial design

The factorial design was carried out using guano samples collected from Gruta do Arnoud’s cave (RN) with the objective of identifying the digestion conditions with the lowest values of dissolved organic carbon (DOC) and residual acidity (RA). These parameters were used as indicators of the efficiency of the digestion process. In addition, the efficiency of the digestion was preliminarily assessed by visual inspection of the resulting solutions, considering the absence of coloration as indicative of effective organic matter decomposition. After digestion, the presence of decanted residual material was also observed and qualitatively identified as undigested silicate phases.

A 2^3^ factorial design with three central point repetitions, conducted in triplicate, was developed and analyzed using STATGRAPHICS Centurion XV.II, resulting in a total of 33 randomized trials. The experimental variables included: HNO_3_ concentration (0.5, 1.0, and 1.5 mol L⁻^1^); digestion time (15, 25, and 35 min); and H_2_O_2_ volume (1, 2, and 3 mL).

The trials were analyzed by ICP OES, and dissolved organic carbon was determined using a calibration curve prepared with a citric acid solution, within a concentration range of 10 to 2000 mg L⁻^1^ of carbon. Additionally, residual acidity was determined by acid–base titration using a standardized 0.1 mol L⁻^1^ NaOH solution.

#### Principal component analysis (PCA)

Principal component analysis (PCA) was employed to evaluate similarities and differences among the guano samples based on metal concentrations. To achieve this, the analysis was conducted separately for each regional dataset (RN and PA) to investigate internal patterns and variability within each region.

For the analysis of the RN data, the matrix was composed of six variables representing the average concentrations (*n* = 3) of the analytes Cd, Co, Cr, Cu, Ni and Pb. The 27 samples from Rio Grande do Norte were arranged in rows. Similarly, a distinct PCA was performed for the PA dataset, using the same six variables, with the 24 samples arranged in rows.

Prior to PCA, the data were autoscaled (mean-centered and scaled to unit variance) and the analysis was performed using MATLAB® software (version 2021a), based on the correlation matrix. Confidence ellipses at the 95% level were included to represent the overall dispersion of the samples. The interpretation focused on the first three principal components, as they accounted for the largest proportion of the explained variance. Score plots were examined using PC1 versus PC2 and PC1 versus PC3, since PC1 represents the main source of variance in the dataset.

## Results

The visual analysis of the digests at different concentrations of HNO_3_ revealed that all concentrations resulted in clear and transparent solutions, with the presence of decanted material identified as undigested silicates. Therefore, in order to minimize the amount of acid used in this procedure, a concentration of 1 mol L⁻^1^ HNO_3_ was chosen as the central point for the factorial design, as this condition demonstrated efficient digestion with a lower volume of reagent employed.

### Factorial design

The DOC and RA results for all experiments are presented in Table 3. Lower DOC values were obtained at higher HNO₃ concentrations (1.5 mol L⁻^1^), longer digestion times (35 min), and higher H₂O₂ volumes (3 mL), while residual acidity increased with increasing acid concentration, remaining within acceptable limits across the experimental conditions.

**Table 3:** Matrix of the full factorial design applied to the microwave-assisted acid digestion step of bat guano samples, and responses in dissolved organic carbon (DOC) and residual acidity (RA)

Factors			(-)	(0)	(+)
**[HNO**_**3**_**]**	HNO_3_ concentration (mol L^−1^)		0.5	1.0	1.5
**t**_**d**_	Digestion time (min)		15	25	35
**V**_**H2O2**_	Volume of H_2_O_2_ (mL)		1	2	3
Experiment	[HNO_3_]	t_d_	V_H2O2_	DOC (mg L^−1^)	AR (mol L^−1^)
1	-	-	-	617.1 ± 212.7	0.64 ± 0.02
2	+	-	-	84.7 ± 24.3	1.89 ± 0.06
3	-	+	-	373.1 ± 213.6	0.59 ± 001
4	+	+	-	35.4 ± 20.4	1.93 ± 0.02
5	-	-	+	288.7 ± 44.8	0.63 ± 0.01
6	+	-	+	211.5 ± 54.3	1.94 ± 0.05
7	-	+	+	350.4 ± 204.0	0.62 ± 0.02
**8**	** + **	** + **	** + **	**13.5 ± 25.5**	**2.01 ± 0.01**
9	0	0	0	278.5 ± 105.0	1.44 ± 0.01
10	0	0	0	405.5 ± 212.4	1.44 ± 0.02
11	0	0	0	92.9 ± 26.6	1.41 ± 0.03

### Validation method

The LOD and LOQ values obtained for the analyzed metals are presented in Table 4. Accuracy assessment, performed through the analysis of the certified reference material (CRM, Solo RM-Agro E2002a), showed agreement between the determined and certified values for most elements. However, Mn exhibited a low recovery (37%). For Co and Ni, accuracy was assessed by addition and recovery tests, with results also presented in Table 4, showing recoveries close to 100% at the different concentration levels. For Zn, inconsistent results were obtained in the performed tests.

**Table 4:** Values of merit of the analytical method for quantification of metals in bat guano (dry mass basis)

Element	Analytical curve^a^	r	LOD^b^ (mg kg^-1^)	LOQ^b^ (mg kg^-1^)	CRM, Soil RM-Agro E2002a		
					Determined values (mg kg^−1^)	Certified values (mg kg^−1^)	Recovery (%)
Cd	A = 155711C – 272.9	0.9998	0.002	0.005	113.4 ± 10.0	94.0 ± 11.4	120 ± 10
Cr	A = 143103C + 5502.7	0.9999	0.001	0.004	0.094 ± 0.004^c^	0,12 ± 0.03^c^	78 ± 5
Cu	A = 299055C – 5988.1	0.9998	0.002	0.007	8.2 ± 0.4	8.8 ± 4.0	93 ± 5
Mn	A = 1000000C – 11353	0.9998	0.001	0.003	0.046 ± 0.002^c^	0.13 ± 0,06^c^	35 ± 1
Pb	A = 6913.8C + 123.28	0.9995	0.006	0.019	214.4 ± 20.7	173.8 ± 18.8	123 ± 11
Element	Analytical curve^a^	r	LOD^b^ (mg kg^-1^)	LOQ^b^ (mg kg^-1^)	Recovery tests at three concentration levels		
					Added concentration (mg L^−1^)	Found concentration (mg kg^−1^)	Recovery (%)
Co (RN-02)	A = 73162C + 283.0	0.9999	0.004	0.011	0	0.26 ± 0.01	--
					2.5	2.84 ± 0.03	103 ± 3
					5.0	5.29 ± 0.01	100 ± 4
					7.5	7.51 ± 0.03	97 ± 2
Ni (RN-02)	A = 51442C + 107.5	0.9997	0.015	0.044	0	0.18 ± 0.03	**--**
					2.5	3.25 ±0.03	122 ± 1
					5.0	5.76 ± 0.02	111 ± 3
					7.5	8.01 ± 0.3	104 ±5
					0	10.6 ± 0.2	
Zn	A = 22983C + 1087.9	0.9999	0.002	0.007	2.5	0.94 ± 0.01	–396 ± 9
					5.0	0.95 ± 0.01	–193 ± 5
					7.5	0.096 ± 0.01	–128 ± 6

^a^*A* analytical signal; *C* concentration, in mg L^-1^

^b^LOD and LOQ: 250 mg sample mass and 15 mL final volume of the digested samples

^c^Concentrations in g kg^-1^

At the end of the validation step of the proposed method, the elements Cd, Cr, Cu and Pb showed satisfactory results for all evaluated performance parameters, with recovery values within the acceptable range and adequate sensitivity. Co and Ni also exhibited satisfactory performance in the recovery tests and were considered validated in terms of accuracy. On the other hand, Mn and Zn did not meet the established criteria and were therefore not considered validated under the experimental conditions employed.

### Elemental analysis of digested guano

Once the optimized digestion condition was established, digestions of the 51 guano samples from both states (RN and PA) were carried out. The sample decomposed were analyzed for Cd, Co, Cu, Cr, Ni, and Pb using ICP OES (Tables 5 and 6).

**Table 5:** Elemental composition of bat guano samples, collected in Rio Grande do Norte, Brazil. Samples were analyzed by ICP OES after microwave-assisted digestion using HNO_3_ and H_2_O_2_. Values are expressed as mean ± standard deviation (mg kg⁻^1^) on a dry mass basis

Sample	Cd	Co	Cr	Cu	Ni	Pb
1	ND	3.25 ± 0.13	22.8 ± 0.6	96.1 ± 4.7	14.9 ± 0.5	7.10 ± 0.25
2	0.37 ± 0.01	1.53 ± 0.04	3.00 ± 0.11	131 ± 3	1.01 ± 0.2	1.19 ± 0.10
3	0.36 ± 0.10	2.93 ± 0.40	7.76 ± 1.23	282 ± 40	6.50 ± 1.15	2.73 ± 0.87
4	0.55 ± 0.02	1.83 ± 0.14	3.62 ± 0.12	175 ± 6	1.27 ± 0.17	1.90 ± 0.10
5	0.71 ± 0.02	1.84 ± 0.02	3.82 ± 0.16	188 ± 5	1.18 ± 0.25	2.73 ± 0.15
6	0.78 ± 0.10	3.20 ± 0.53	8.26 ± 1.42	271 ± 40	4.14 ± 0.70	2.71 ± 0.60
7	0.66 ± 0.30	1.66 ± 0.26	3.00 ± 0.38	121 ± 14	1.55 ± 0.18	0.88 ± 0.35
8	0.28 ± 0.03	4.46 ± 0.53	15.5 ± 2.1	172 ± 17	16.2 ± 1.7	4.20 ± 0.14
9	0.67 ± 0.06	2.59 ± 0.38	10.3 ± 1.3	240 ± 30	3.82 ± 0.80	4.48 ± 0.83
10	0.30 ± 0.03	1.46 ± 0.15	4.84 ± 0.34	169 ± 5	6.00 ± 0.71	2.25 ± 0.60
11	0.39 ± 0.15	0.47 ± 0.04	3.00 ± 0.27	121 ± 2	0.39 ± 0.30	1.17 ± 0.50
12	0.55 ± 0.05	6.74 ± 0.60	31.8 ± 2.3	65.0 ± 3.0	20.6 ± 1.28	39.1 ± 2.0
13	0.51 ± 0.03	2.03 ± 0.06	10.5 ± 0.1	145 ± 2	10.4 ± 0.1	2.24 ± 0.36
14	0.44 ± 0.03	0.82 ± 0.01	3.34 ± 0.45	230 ± 17	1.50 ± 0.20	0.60 ± 0.32
15	0.53 ± 0.20	0.34 ± 0.07	6.00 ± 0.20	152 ± 3	1.18 ± 0.44	2.10 ± 0.50
16	ND	1.79 ± 0.06	6.30 ± 0.30	153 ± 8	2.87 ± 0.07	1.78 ± 0.26
17	0.87 ± 0.04	3.32 ± 0.30	7.70 ± 0.73	333 ± 34	6.71 ± 1.57	2.62 ± 0.04
18	0.88 ± 0.46	2.92 ± 0.10	22.1 ± 2.6	111 ± 6	16.4 ± 1.2	5.60 ± 1.10
19	0.38 ± 0.01	1.52 ± 0.07	6.90 ± 0.11	173 ± 4	6.40 ± 0.17	1.57 ± 0.14
20	2.32 ± 0.09	5.19 ± 0.30	18.6 ± 2.1	186 ± 9	11.9 ± 1.1	30.7 ± 2.8
21	ND	1.40 ± 0.08	8.07 ± 0.70	167 ± 9	4.84 ± 0.14	2.24 ± 0.63
22	0.32 ± 0.02	2.23 ± 0.66	18.0 ± 1.9	102 ± 2	10.2 ± 1.5	5.30 ± 1.25
23	0.28 ± 0.01	0.12 ± 0.03	1.61 ± 0.05	82.7 ± 1.2	ND	0.30 ± 0.11
24	0.33 ± 0.01	0.52 ± 0.03	3.70 ± 0.13	204 ± 3	0.87 ± 0.14	1.31 ± 0.25
25	0.65 ± 0.17	3.41 ± 0.54	9.84 ± 2.00	249 ± 42	4.41 ± 0.63	2.50 ± 0.55
26	0.71 ± 0.08	1.66 ± 0.22	4.47 ± 1.16	283 ± 43	1.53 ± 0.45	1.58 ± 0.24
27	0.40 ± 0.07	2.80 ± 0.53	7.11 ± 2.00	215 ± 46	4.43 ± 1.40	1.50 ± 0.71

*ND* not detected

**Table 6:** Elemental composition of bat guano samples, collected in Pará, Brazil. Samples were analyzed by ICP OES after digestion assisted by microwave radiation using HNO_3_ and H_2_O_2_. Values are expressed as mean ± standard deviation (mg kg⁻^1^) on a dry mass basis

Sample	Cd	Co	Cr	Cu	Ni	Pb
1	1.14 ± 1.12	ND	ND	15.1 ± 2.1	1.00 ± 0.31	2.04 ± 0.88
2	ND	ND	ND	10.4 ± 1.9	0.60 ± 0.07	1.67 ± 0.07
3	0.57 ± 0.01	ND	ND	213 ± 6	1.24 ± 0.07	3.08 ± 0.07
4	1.05 ± 0.06	ND	ND	285 ± 18	0.72 ± 0.08	2.08 ± 0.50
5	0.70 ± 0.04	ND	0.44 ± 0.10	158 ± 3	1.00 ± 0.11	2.01 ± 0.20
6	0.70 ± 0.01	ND	1.24 ± 0.10	220 ± 3	0.52 ± 0.17	2.40 ± 0.20
7	ND	ND	1.20 ± 0.20	121 ± 30	1.63 ± 0.10	20.0 ± 1.67
8	ND	ND	0.84 ± 0.14	93.8 ± 24.0	1.91 ± 0.15	19.4 ± 1.6
9	ND	ND	ND	34.4 ± 2.6	ND	1.21 ± 0.14
10	0.52 ± 0.01	ND	ND	365 ± 9	2.02 ± 0.01	ND
11	ND	ND	ND	260 ± 15	1.14 ± 0.24	4.26 ± 0.48
12	ND	ND	ND	23.0 ± 0.3	ND	ND
13	ND	ND	ND	29.9 ± 0.7	ND	ND
14	ND	ND	ND	87.4 ± 6.8	2.10 ± 0.22	ND
15	0.77 ± 0.08	ND	ND	198 ± 28	ND	1.22 ± 0.25
16	ND	ND	ND	226 ± 8	0.94 ± 0.05	0.80 ± 0.01
17	ND	ND	ND	280 ± 11	0.97 ± 0.07	1.13 ± 0.01
18	ND	ND	ND	69.0 ± 3.3	1.28 ± 0.20	ND
19	ND	ND	ND	70.1 ± 5.5	1.80 ± 0.04	0.90 ± 0.05
20	ND	ND	1.10 ± 0.17	215 ± 16	ND	13.3 ± 0.4
21	ND	ND	ND	65.1 ± 4.5	1.05 ± 0.12	2.90 ± 0.80
22	ND	ND	ND	32.0 ± 4.1	ND	ND
23	0.97 ± 0.05	ND	0.33 ± 0.27	646 ± 61	2.10 ± 0.11	3.92 ± 0.33
24	ND	ND	ND	12.3 ± 1.4	ND	0.67 ± 0.13

*ND* not detected

### Vertical distribution of metals in the guano of insectivorous bats (*Pteronotus gymnonotus*) in the Furna do Urubu cave

The analysis of toxic metal concentrations in bat guano collected at different depths in the Furna do Urubu cave revealed distinct patterns across the deposit layers (Table 7). The analyzed material originates predominantly from bats of the genus *Pteronotus gymnonotus*, whose diet is primarily insectivorous. Elements such as Co, Cr, Ni, and Pb were at higher concentrations at intermediate depths, without a clear linear trend. Cd exhibited a relatively constant distribution throughout the profile, suggesting uniform deposition of this metal in the cave environment. In contrast, there was a progressive increase in the concentrations of Cu with increasing depth of the deposit.

**Table 7:** Elemental composition of bat guano samples from *Pteronotus gymnonotus*, collected in Furna do Urubu cave, Rio Grande do Norte, Brazil. Samples were analyzed by ICP OES, after microwave radiation-assisted digestion using HNO_3_ and H_2_O_2_. Values are expressed as mean ± standard deviation (mg kg⁻^1^) on a dry mass basis

Sample depth (cm)	Cd	Co	Cr	Cu	Ni	Pb
5 (12–14)	0.7 ± 0.1	1.8 ± 0.1	3.8 ± 0.2	187.7 ± 4.9	1.2 ± 0.3	2.7 ± 0.2
25 (28–30)	0.65 ± 0.2	3.4 ± 0.5	9.8 ± 2.0	248.9 ± 42.0	4.4 ± 0.6	2.5 ± 0.6
27 (44–46)	0.4 ± 0.1	2.8 ± 0.5	7.1 ± 2.0	215.1 ± 45.6	4.4 ± 1.4	1.5 ± 0.7
6 (58–60)	0.8 ± 0.1	3.2 ± 0.5	8.3 ± 1.4	271.2 ± 40.5	4.1 ± 0.7	2.7 ± 0.6
9 (76–78)	0.67 ± 0.06	2.6 ± 0.4	10.3 ± 1.4	240.0 ± 30.1	3.8 ± 0.8	4.5 ± 0.8
3 (96–98)	0.4 ± 0.1	2.9 ± 0.4	7.8 ± 1.2	281.6 ± 40.0	6.5 ± 1.2	2.7 ± 0.9
17 (120–122)	0.87 ± 0.04	3.3 ± 0.3	7.7 ± 0.7	332.7 ± 34.4	6.7 ± 1.6	2.6 ± 0.04
26 (148–150)	0.7 ± 0.1	1.7 ± 0.2	4.5 ± 1.2	283.0 ± 43.3	1.6 ± 0.2	1.6 ± 0.2

### Principal component analysis (PCA)

**Fig. 2:**
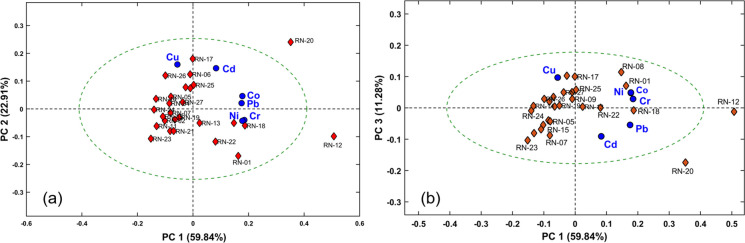
Principal component analysis describing the distribution of guano samples from the state of Rio Grande do Norte (RN), Brazil. (**a**) PC1 × PC2 and (**b**) PC1 × PC3. The guano samples (scores) are represented by red rhombuses, the metals (loadings) by blue circles. The dashed circle corresponds to the 95% confidence ellipse

For the guano samples from Rio Grande do Norte, the analysis revealed that three principal components explained 94.0% of the total variance (Fig. 2). The individual contributions were: PC1 accounted for 59.8%, PC2 for 22.9%, and PC3 for 11.3%. In the PC1 × PC2 score plot (Fig. 2a), PC1 was strongly influenced by Cr, Ni and Pb, whereas PC2 was mainly associated with Cd, Co and Cu. These metal associations may reflect a combination of anthropogenic inputs and natural geochemical signatures related to the cave lithology. The PC1 × PC3 projection (Fig. 2b) indicated a strong correlation between PC3 and the elements Cd, Cr, Cu, Ni and Pb. The dashed circle corresponds to the 95% confidence ellipse.

The multivariate distribution of samples allowed the identification of at least three distinct clusters, in addition to a few outlying samples:

- *Group 1—*This group includes samples with generally low concentrations of most analyzed metals (e.g., RN-02, RN-07, RN-10, RN-11, RN-15, RN-19, RN-21 and RN-23). These samples originated from three different sites—Gruta dos Pingos, Cavernas Furna Feia and Furna do Urubu—and are associated with guano from insectivorous bats.
- *Group 2—*Includes samples RN-01, RN-08, RN-13, RN-18 and RN-22, characterized by elevated concentrations of Cr and Ni. These samples were collected from Furna Feia Cave and correspond to guano from insectivorous (*Pteronotus gymnonotus*) and omnivorous (*Phyllostomus discolor*) bats.
- *Group 3—*Comprises samples with high levels of Cu, such as RN-03, RN-06, RN-09, RN-17, RN-25, and RN-26. All samples were collected from Furna do Urubu Cave and are associated with insectivorous bat guano.
- *Isolated Samples—*Samples RN-12 and RN-20 showed the highest scores along PC1, indicating elevated metal concentrations. Sample RN-12 was mainly characterized by high levels of Cr and Pb, while RN-20 exhibited elevated concentrations of Cd, Cr, and Pb. Both samples were collected from Gruta do Arnoud, with RN-12 originating from nectarivorous bats and RN-20 from insectivorous bats. Sample RN-20 displayed atypical behavior in the PCA plots, with concentrations of Cd (2.3 mg·kg⁻^1^), Cr (18.6 mg·kg⁻^1^), and Pb (30.7 mg·kg⁻^1^). Similarly, sample RN-12 also presented high values, with Cr (31.8 mg·kg⁻^1^) and Pb (39.1 mg·kg⁻^1^).

**Fig. 3:**
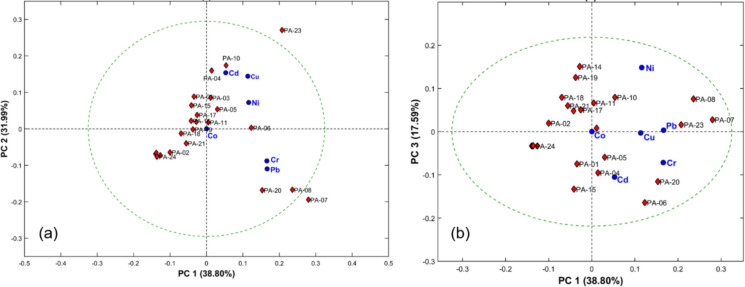
Principal component analysis describing the distribution of guano samples from the state of Pará (PA), Brazil. **a** PC1 × PC2 and (**b**) PC1 × PC3. The guano samples (scores) are represented by red rhombuses, the metals (loadings) by blue circles. The dashed circle corresponds to the 95% confidence ellipse

For the guano samples from Pará, PCA analysis indicated that three principal components explained 88.4% of the total variance. The contributions were: PC1 with 38.8%, PC2 with 32.0%, and PC3 with 17.6%. In the PC1 × PC2 plot (Fig. 3a), PC1 is mainly associated with Cr and Pb, whereas PC2 shows a stronger association with Cd, Cu, and Ni. Co presents a lower contribution compared to the other elements. In the PC1 × PC3 projection (Fig. 3b), PC3 is mainly associated with Cd, Cr and Ni contribute in the opposite direction along this component, as indicated by the orientation of the vectors.

Based on the distribution of samples in the PC1 × PC2 and PC1 × PC3 biplots, three distinct groups were revealed based on the similarity of their metal profiles:

- *Group 1—*Exemplified by samples PA-02, PA-09, PA-21 and PA-24, collected from Caverna das Mãos (frugivorous bats), Chico Preta (frugivorous bats), Planaltina (omnivorous bats) and Pedra da Cachoeira (insectivorous bats), exhibited low concentrations of most analyzed elements. This suggests these areas have minimal anthropogenic impact or are regions with naturally low levels of trace metals.
- *Group 2—*Includes samples such as PA-03, PA-04, PA-05, PA-10, and PA-23, collected from Caverna das Mãos, Coração da Amazônia, and Planaltina, all associated with guano from insectivorous bats, showed elevated concentrations of Cu, Cd and Ni. The sample PA-23 stands out due to its higher Cd content, positioning itself outside the confidence ellipse, which indicates a profile that deviates from the other samples.
- *Group 3—*Samples such as PA-07, PA-08, and PA-20, from the Chico Preta and Planaltina caves, associated with guano from insectivorous bats, were characterized by high levels of Cr and Pb. It is worth noting that sample PA-07 is located beyond the boundaries of the confidence ellipse, highlighting the strong influence of o Cr (1.20 mg·kg⁻^1^) e do Pb (20.0 mg·kg⁻^1^) on its composition, compared to the other samples.

The PCA also revealed that Co appeared close to the origin of the plots, suggesting it does not contribute significantly to the total variance. This indicates very low or negligible levels of Co contamination in the region.

## Discussion

### Study area

Understanding the environmental and socioeconomic characteristics of the sampled areas is essential for interpreting the patterns of metal concentrations observed in guano. The caves sampled in the state of Pará are located within the Amazon biome and are distributed along the Trans-Amazonian Highway. This region has undergone intense processes of habitat loss and fragmentation, with the surrounding matrix of the cave shelters being transformed from predominantly forested landscapes into highly anthropized areas, mainly due to agricultural expansion, particularly cocoa cultivation. In addition, the study area is located near the Carajás region, an important economic and mining hub, recognized as the largest iron ore complex in Brazil and also hosting the country’s largest caves developed in iron-rich formations (ICMBio, 2016). The predominant lithology in the region is sandstone. In general, the Amazonian climate is characterized by high rainfall and persistently elevated temperatures, with annual values ranging from 22.0 to 31.5 °C (Scerne, 2001).

In contrast, the caves sampled in the state of Rio Grande do Norte are located within the Brazilian semi-arid Caatinga domain, where mean annual temperatures range from 25 to 30 °C and average annual precipitation varies between 600 and 1000 mm (Silva et al., 2017). The main economic activities in this region include agribusiness, tourism, and mining, with particular emphasis on marine salt extraction, which accounts for approximately 90% of national production, as well as the exploitation of limestone, tin, natural gas, petroleum, and feldspar. The state also stands out for its renewable energy production, especially wind power (Moura et al., 2019). Unlike the study area in Pará, the predominant lithology in the sampled municipalities of Rio Grande do Norte is limestone.

The selection of Cd, Cu, Cr, Ni, Pb, Mn, and Zn for analysis was guided by the environmental, geological, and socioeconomic characteristics of the cave surroundings described above. Elements such as Cu, Mn, Zn, Cr, and Ni are commonly associated with natural geochemical backgrounds, reflecting local lithology, soil composition, and weathering processes, particularly in regions dominated by sandstone and limestone formations (Gloaguen & Passe, 2017; Zhang et al., 2025). In contrast, Cd and Pb are widely recognized as non-essential and potentially toxic elements, frequently linked to anthropogenic inputs related to agricultural practices, mining activities, and land-use change, which are prominent in both study regions. Thus, the combined evaluation of essential and non-essential metals allows the discrimination between geogenic controls and potential external contributions, providing a more robust framework for interpreting spatial variability in metal concentrations in guano across contrasting cave environments (Forray et al., 2024).

### Factorial design

Based on the factorial design, the effectiveness of the digestion conditions can be discussed in terms of their influence on DOC and residual acidity. Higher acid concentrations, longer digestion times, and increased H₂O₂ volumes were associated with lower DOC values, indicating a more efficient decomposition of the organic matrix. However, residual acidity tended to increase with increasing acid concentration, although the observed values remained within acceptable limits. Overall, the results indicate that even under more acidic conditions, the digestion procedure was effective in minimizing residual organic matter while maintaining controlled acid residues.

### Validation method

The validation of analytical methods is an essential step to ensure the reliability of the obtained results. The data presented in Table 4 demonstrated excellent linearity, with correlation coefficients (r) higher than 0.999 for all elements, meeting the acceptance criteria (*r* ≥ 0.995) and confirming the suitability of the linear model within the studied range.

The low LOD and LOQ values indicate high method sensitivity, allowing the determination of trace metals even in a complex matrix such as guano. Accuracy, evaluated using a certified reference material, showed acceptable recoveries for Cd (120%), Cr (82%), and Cu (98%), within the 80–120% range (US Epa, 2018). In contrast, Pb (123%) presented a value above the upper limit, suggesting possible interferences or matrix effects. Mn and Zn exhibited unsatisfactory recoveries (35% for Mn and negative values for Zn), indicating significant limitations of the method for these elements, particularly due to possible matrix effects and analytical interferences affecting Zn quantification.

For Co and Ni, evaluated through recovery (spike) tests, recoveries ranged from 97–103% (Co) and 104–122% (Ni), remaining within the acceptable limits for spiked samples (75–125%), confirming the accuracy of the method for these analytes. Precision was satisfactory, with low deviations, indicating good reproducibility (Silva et al., 2020; US Epa, 2018).

Overall, although the method showed adequate performance for most elements, Mn and Zn did not meet the validation criteria and were therefore excluded from subsequent analyses.

### Elemental analysis of digested guano

A general analysis of the trace element concentrations, determined by ICP OES, indicated that Cu was abundantly present in the guano samples from both Rio Grande do Norte and Pará. In the guano from RN, concentrations ranged between 65.0 and 333 mg kg⁻^1^ and, in the guano from PA, ranged between 10.4 and 646 mg kg⁻^1^. Similar findings were reported by (Wurster et al., 2015) who studied bat guano from five different caves in Palawan and Malaysia, and found elevated concentrations of Cu, Cr, and Zn in all samples. According to that authors, those concentrations resulted due to the bat’s diet, which likely contained contaminated food sources. Additionally, bioaccumulation of Cu and Zn at higher concentrations in guano may occur because these metals are essential micronutrients commonly present in soil and have a key role in plant growth and root development. Copper acts as a vital redox-active cofactor in key physiological processes, including photosynthesis, respiration and cell wall lignification; its deficiency leads to stunted growth, leaf chlorosis, and reproductive impairment (Lilay et al., 2024). Zinc, for its part, plays a regulatory role in enzymes critical for chlorophyll, protein, carbohydrate synthesis, and auxin metabolism; its deficiency impairs root development, reduces leaf size, and results in chlorosis and growth inhibition (Biosci et al., 2024). Therefore, bat species feeding directly on plant materials (frugivores and nectarivorous) or on insects (insectivorous) would be exposed to such elements. In addition to dietary factors, local environmental conditions and natural geochemical characteristics of the surrounding areas may also influence the baseline levels of these elements in guano.

Regarding the guano samples from Rio Grande do Norte, sample 20 (Table 5)—obtained from Gruta do Arnoud, referring to surface guano and insectivorous bats of the species *Pteronotus gymnonotus*—exhibited elevated concentrations of Cd and Pb (2.32 and 30.7 mg kg⁻^1^, respectively) compared to other samples from the same region. Similarly, sample 12 (Table 5)—obtained from Gruta do Arnoud, referring to surface guano and nectivorous bats of the species *Glossophaga soricina*—showed higher levels of Co, Cr, Ni and Pb (6.74, 31.8, 20.6 and 39.1 mg kg⁻^1^, respectively) compared to the other samples from RN. These bat species have distinct dietary habits: *G. soricina* is nectivorous, feeding mainly on nectar, while *P. gymnonotus* is insectivorous, feeding primarily on insects. Toxic metal contamination may differ between these dietary types (Giunta et al., 2024). Nectarivorous bats are primarily exposed to toxic metals through the ingestion of contaminated nectar and/or pollen, particularly in areas affected by industrial and agricultural activities. Additionally, these bats may consume insects associated with flowers, as well as contaminated water sources (Folador et al., 2023). On the other hand, insectivorous bats tend to accumulate toxic metals through biomagnification in the food chain, with metals being transferred from sediments, water, soil, and plants to insects, and subsequently to the bats (Folador et al., 2022; Wurster et al., 2015). The elevated levels of Cd, Co, Cr, and Pb observed in the guano samples from RN may reflect a combination of environmental influences, considering that these caves are located within a semi-arid region characterized by intense agricultural activity and diverse mining operations, including limestone, tin, oil, and marine salt extraction. In addition, the predominance of limestone lithology in the sampled municipalities may contribute to the local geochemical background of these elements. Therefore, when compared to other locations, these findings suggest plausible regional influences on metal occurrence, rather than allowing a direct attribution to anthropogenic contamination alone.

For samples from Pará, samples 7 and 8—obtained from Chico Preta cave, referring to surface guano and insectivorous bats of the species *Natalus macrourus*, *Pteronotus rubiginosus/P. alitonus*—exhibited elevated Pb concentrations, with values of 20.0 and 19.4 mg kg⁻^1^, respectively, compared to other samples from the same locality. In contrast, sample 9—obtained from Chico Preta cave, referring to surface guano and frugivorous bat of the species *Carollia perspicillata*—showed a lower Pb concentration of 1.21 mg kg⁻^1^. The variation can be explained by bioaccumulation. Metals present in the soil, water, and plants accumulate in insects, which are then transferred to insectivorous bats through the food chain (Zukal et al., 2015). This process may be further influenced by regional environmental conditions, as the sampled caves are located in an Amazonian area subject to intense land-use change associated with agricultural expansion along the Trans-Amazonian Highway and proximity to the Carajás mining region. In contrast, the guano from frugivorous bats, which are less exposed to this type of bioaccumulation, showed a lower concentration of lead, suggesting that dietary pathways play a primary role, while regional anthropogenic and geochemical factors may act as additional contributing influences.

In Brazil, there is no specific legislation that establishes maximum tolerable limits for contaminants present in bat guano. Therefore, the results obtained in this study were compared with metal concentrations reported in the scientific literature for guano samples (Gallant et al., 2020; Wurster et al., 2015; Zukal et al., 2015). The concentration ranges are presented for descriptive and comparative purposes only. No inferential statistical tests were applied, and therefore the observed differences between regions are interpreted as indicative patterns of variability rather than statistically significant differences.

A relevant example is the study by Gallant et al. (2020), which analyzed a guano deposit in Jamaica to investigate temporal variations in toxic metal concentrations, highlighting environmental changes associated with the Industrial Revolution. The authors reported significant increases in the concentrations of metals such as Cd, Pb, Hg and Zn, especially after the Industrial Revolution period. In particular, the concentrations of Cd and Pb reached values around 92.8 mg kg⁻^1^ and 61–65 mg kg⁻^1^, respectively, which surpassed those found here. Such elevated concentrations were attributed to intense emissions of toxic metals from industrial activities, such as mining, coal combustion, and waste incineration, which were prevalent during that era. Additionally, the high levels of Cd detected in that study may also be partially explained by the local geology context, as approximately 67% of Jamaican soils are covered by limestone, a material often associated with phosphate minerals rich in Cd, such as phosphorite, which can contain concentrations as high as 6000 mg kg⁻^1^ of Cd, further contributing to the elevated values observed.

Zukal et al (2015), in a review of 52 studies reporting on heavy metal concentrations in bats, observed that frugivores and nectarivorous tend to have higher concentrations of Cd, Co, Cr, Cu, Ni, Pb, and Zn in their guano compared to insectivorous bats. This difference is attributed to the distinct exposure pathways to toxic metals between dietary groups, as previously discussed. For insectivorous bats, the reported elemental concentration ranges (in mg kg⁻^1^) were: 0.03–8.5 (Cd); < 2 (Co); 0.5–57 (Cr); 40–2869 (Cu); < 1–16 (Ni); 0.52–65 (Pb) and 64–1079.83 (Zn) (Zukal et al., 2015). These values provide a useful reference for comparing metal contamination across different bat species and ecological contexts.

Based on a comparison of our results with the literature, the metal concentrations in our study align with the observations of Zukal et al. (2015), which highlight the importance of bat diet in bioaccumulation. The metal concentrations in guano samples from insectivorous and nectarivorous bats fit within the reference ranges, reinforcing that bioaccumulation through the food chain is a determining factor. While the concentrations of Cu are elevated, they are comparable to those found by Wurster et al. (2015) and can be explained by their presence as essential micronutrients. However, these concentrations are not attributed exclusively to physiological requirements, as local environmental conditions and regional land-use characteristics may also influence metal availability and uptake. In contrast to the study by Gallant et al. (2020), which reported significantly higher Cd and Pb concentrations due to intense industrial contamination from the Industrial Revolution, our lower values suggest that the areas studied in Brazil were not exposed to the same degree of historical pollution. Thus, the observed metal levels likely reflect a combination of dietary exposure pathways and site-specific environmental and geochemical factors, rather than definitive evidence of intense anthropogenic contamination. Such comparisons with the literature, in the absence of specific Brazilian legislation, provide a valuable context for assessing potential heavy metal contamination in the studied areas.

Beyond the environmental assessment, these findings also raise concerns regarding biota exposure. Although metal concentrations in guano do not directly reflect internal tissue burdens, they provide valuable insight into dietary exposure pathways and metal assimilation processes. Importantly, the proportion of metals eliminated via feces is metal-dependent. Essential elements such as Cu and Zn are subject to physiological homeostatic regulation, with absorption and retention adjusted according to metabolic demand, and excess amounts being excreted. In contrast, non-essential metals such as Pb and Cd have no biological function and tend to be either excreted more readily or sequestered in specific tissues, such as bones and kidneys, resulting in distinct fecal excretion patterns (Sandoval-herrera et al., 2023; Zukal et al., 2015).

Chronic exposure to non-essential elements or excessive levels of essential metals may induce sub-lethal physiological effects in bats, including oxidative stress, neurochemical alterations, and renal damage. However, because toxicity thresholds for bats under natural conditions remain poorly defined, the concentrations reported here should be interpreted as indicators of potential exposure rather than definitive evidence of toxicity. Given bats’ high metabolic rates, longevity, and trophic position, sustained exposure to these metals may contribute to cumulative physiological stress over time (Vidal et al., 2024; Zukal et al., 2015). In Neotropical species, the direct linkage between environmental metal exposure and specific health outcomes remains a major knowledge gap in ecotoxicology, reinforcing the role of guano as a non-invasive indicator of environmental contamination along trophic pathways, while attribution to specific anthropogenic sources should be approached with caution (Vidal et al., 2024).

### Vertical distribution of metals in the guano of insectivorous bats (*Pteronotus gymnonotus*) in the Furna do Urubu cave

**Fig. 4:**
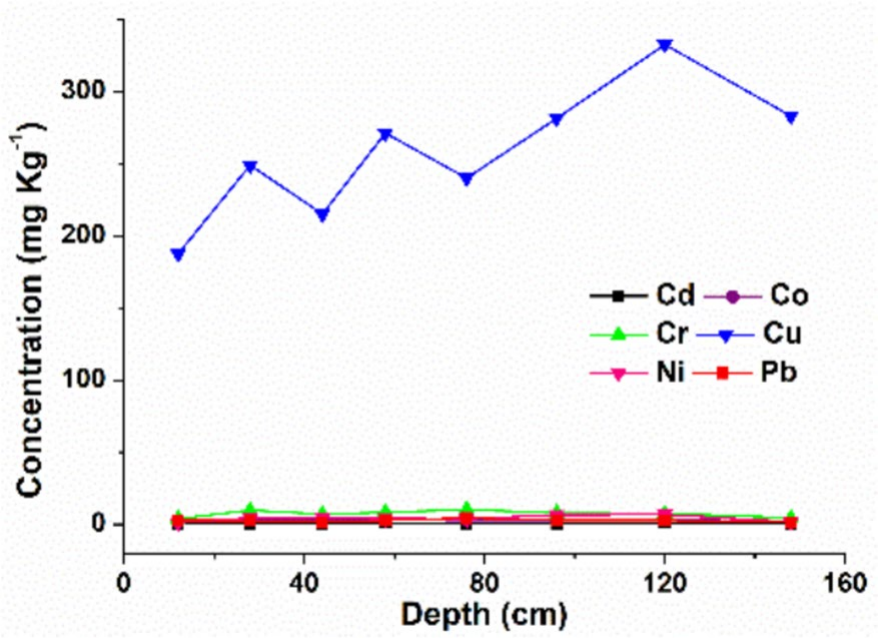
Vertical distribution of metal concentrations in guano samples of insectivorous bats (*Pteronotus gymnonotus*) from different depths in the Furna do Urubu cave, Rio Grande do Norte, Brazil. The legend shows that the identified elements are: Cd (black square); Cr (green triangle); Co (purple circle); Cu (blue triangle); Ni (pink triangle) and Pb (red square)

Based on the patterns identified in the vertical distribution of metals, it is possible to discuss the factors that may have influenced these variations throughout the guano profile (Fig. 4).

The observed increase in Cu in deeper layers suggests greater historical deposition or better preservation of these elements in older guano layers, possibly linked to past environmental variations or changes in the bats’ diet. Metals such as Cu, while essential in small amounts for biological processes, can become toxic when accumulated at elevated levels, and are often linked to the ingestion of bioaccumulator insects, such as coleopterans and dipterans (Wurster et al., 2015). The constant distribution of Cd throughout the profile may indicate stable and persistent sources over time. Regardless of the potential causes, such as changes in insect fauna composition, seasonal variations, or human activities in the surrounding environment, these results show that insectivorous bat guano can serve as a useful tool for environmental monitoring, especially in sites with well-defined stratification, such as Furna do Urubu. This approach contributes to understanding the ecological history of the area and assessing potential contamination in subterranean environments.

### Principal component analysis (PCA)

The PCA results reveal a heterogeneous distribution of metals in bat guano across caves in both Rio Grande do Norte and Pará, reflecting environmental and anthropogenic influences as well as dietary habits. In RN, samples from Furna Feia and Furna do Urubu, such as RN-02, RN-07, and RN-10, associated with insectivorous bats (*Pteronotus gymnonotus*), exhibited low concentrations of most metals, suggesting areas of minimal exposure to contamination. In contrast, other samples from the same caves, including RN-01, RN-08, and RN-17, showed elevated levels of Cr, Ni, and Cu. This variation within caves may reflect heterogeneous contamination sources or differences in the foraging areas used by bats, with some zones influenced by anthropogenic activities, such as fertilizers, pesticides, and metallic waste, and others more pristine. Isolated samples from the RN region, such as RN-12 and RN-20, both collected from Gruta do Arnoud (nectarivorous and insectivorous bats, respectively), showed elevated metal concentrations. Despite differences in feeding habits, the results suggest that both nectarivorous and insectivorous bats are exposed to similar contamination sources, possibly related to naturally enriched food resources or localized anthropogenic pollution. Sample RN-12 exhibited atypical concentrations of Cr, Ni, and Pb, while sample RN-20 showed elevated levels of Cd, Cr, and Pb. These findings support the presence of localized environmental contamination hotspots capable of affecting organisms with different feeding strategies.

In Pará, PCA analysis revealed more defined grouping patterns, reflecting spatial heterogeneity and trophic influences. Low-metal samples, such as PA-02 (Caverna das Mãos, frugivorous bats), PA-09 (Chico Preta, frugivorous bats), and PA-24 (Pedra da Cachoeira, insectivorous bats), exhibited low concentrations of most metals, indicating minimal anthropogenic impact or naturally low geological metal content. In contrast, samples PA-04 (Caverna das Mãos), PA-10 (Coração da Amazônia), and PA-23 (Planaltina), all from insectivorous bat guano, showed elevated concentrations of Cu and Cd, suggesting bioaccumulation through diet. Similarly, PA-07, PA-08, and PA-20 (Chico Preta and Planaltina caves, insectivorous bats) exhibited high Pb and Cr levels, highlighting the vulnerability of insectivorous bats to metal biomagnification. The negligible contribution of Co, appearing near the origin of the PCA plots, indicates minimal contamination by this element in Pará.

Overall, the integrated analysis demonstrates that bat guano serves as a sensitive indicator of environmental metal distribution. Within both RN and PA, the combination of dietary habits, foraging areas, and cave-specific conditions leads to significant variability in metal accumulation. While RN exhibits higher overall metal concentrations with overlapping groups, PA shows clearer separation between groups, reflecting spatial heterogeneity. These findings underscore the importance of species of bats monitoring for assessing environmental contamination in subterranean ecosystems.

## Conclusion

This study demonstrated that bat guano is a potential bioindicator for assessing the presence of toxic metals in the environment, reflecting both the ecological characteristics of the species and the environmental conditions of the caves where the bats live. The application of the optimized digestion method allowed precise analysis of metals such as Cd, Co, Cr, Cu, Ni, and Pb in samples collected from Rio Grande do Norte (RN) and Pará (PA). Samples from RN showed higher metal concentrations, indicating greater environmental exposure, which may be associated with the semi-arid Caatinga environment, the predominance of limestone lithology, and the presence of regional economic activities such as mining, marine salt extraction, and agribusiness, which are here considered potential anthropogenic influences rather than definitive contamination sources. The analysis of the vertical distribution of elements in the guano from Furna do Urubu revealed distinct patterns, with Cu, increasing with depth, suggesting a deposition history or better preservation in older layers. Elements such as Co, Cr, Ni, and Pb showed intermediate peaks that may reflect specific depositional conditions or environmental variations over time within the cave system. The PCA analysis identified distinct clusters among the samples and highlighted locations with possible localized contamination, such as Gruta do Arnoud (RN) and the Planaltina and Chico Preta caves in Pará, with these patterns interpreted in an exploratory manner. The sampled caves in Pará are located within the Amazon biome, in areas affected by habitat loss and landscape fragmentation linked to agricultural expansion and proximity to the Carajás mining province, characterized by iron-rich geological formations and sandstone lithology. The analysis also emphasized relationships with the dietary habits of the species (insectivorous, frugivorous, nectarivorous, and omnivorous). Overall, these results reinforce the role of guano as an ecological and historical indicator of pollutants, highlighting its applicability for long-term environmental monitoring in cave ecosystems. At the same time, the associations observed between metal concentrations, environmental context, and potential anthropogenic influences should be regarded as indicative and exploratory, pending the integration of independent data on land-use history, regional geochemical baselines, and other external environmental variables, which represent important future perspectives for this type of study.

## Supplementary Information

Below is the link to the electronic supplementary material.

Supplementary file1 (DOCX 16.4 KB)

## Data Availability

No datasets were generated or analyzed during the current study.
