# Supplementary material for: Metal levels in bat guano from Brazilian caves: insights into environmental contamination
Source: Environ Monit Assess. 2026 May 23;198(6):645. doi: 10.1007/s10661-026-15456-1 (PMC13198488; doi:10.1007/s10661-026-15456-1)
Supplement: Supplementary file 1 — Supplementary file1 (DOCX 16.4 KB) [file 10661_2026_15456_MOESM1_ESM.docx]

**Environmental Monitoring and Assessment**

Submission ID: 65f63e61-de39-4184-b610-9e337d66d5ca

**Manuscript:** **Metal Levels in Bat Guano from Brazilian Caves: Insights into Environmental Contamination**

Authors: Karla Beatriz Oliveira da Silva, Narjara Tércia Pimentel, Eder Barbier, Enrico Bernard, Ana Paula Silveira Paim

**Supplementary Material**

**Table S1.** Instrumental conditions and operational parameters for the ICP OES.

| **Parameters** | |
| --- | --- |
| Power RF (kW) | 1.3 |
| Nebulizer gas flow (L min^-1^) | 0.8 |
| Auxiliary gas flow (L min^-1^) | 0.2 |
| Plasma gas flow (L min^-1^) | 15 |
| Sample aspiration rate (mL min^-1^) | 1.0 |
| Flush time (s) | 5 |
| Replicates | 3 |
| Nebulizer | Cross flow |
| Spray chamber | Cyclonic |
| Nebulization chamber | Scott |
| Plasma observation view | Axial |
| Observation height (mm) | 15 |
| Element and wavelength (nm) | C 193.030 (I); Cd 228.802 (I); Co 228.616 (II); Cr 267.716 (I); Cu 327.393 (I); Mn 257.610 (II); Ni 231.604 (II); Pb 220.353 (II); Zn 206.200 (II) |

_(I) and (II) – atomic and ionic emission lines, respectively_.

**Table S2**. Experimental conditions of power, time, and temperature of the microwave oven used for the decomposition of bat guano samples.

| **Power (W)** | **Heating ramp** | **Cooling** |
| --- | --- | --- |
| 800 | 35 min; 190 °C  35 min; 190 °C | 20 min |
